# The Society of Medicine

**Published:** 1888-01

**Authors:** 


THE SOCIETY OF MEDICINE.—At the annual meeting of the Atlanta Society of Medicine, held December 20, 1887, the following officers were elected for the ensuing year:

President—Dr. Virgil O. Hardon.
Vice-President—Dr. W. S. Elkin.
Secretary—Dr. L. H. Jones.
Treasurer.—Dr. E. van Goidtsnoven.
Librarian—Dr. N. O. Harris.
Reporters—Dr. A. B. Ashworth, Dr. W. F. Westmoreland, Jr., Dr. H. P. Cooper.

